# Evaluation of next-generation sequencing versus next-generation flow cytometry for minimal-residual-disease detection in Chinese patients with multiple myeloma

**DOI:** 10.1007/s12672-024-00938-w

**Published:** 2024-03-19

**Authors:** Mo Zhou, Yan Chen, Yanlei Gong, Mingqing Zhu, Jiannong Cen, Jinlan Pan, Lingzhi Yan, Jingjing Shang, Song Jin, Xiaolan Shi, Weiqin Yao, Shuang Yan, Depei Wu, Suning Chen, Chengcheng Fu, Li Yao

**Affiliations:** 1 National Clinical Research Center for Hematologic Diseases, Jiangsu Institute of Hematology, The First Affiliated Hospital of Soochow University, Soochow University, 188 Shizi Street, Suzhou, 215006 People’s Republic of China; 2 Institute of Blood and Marrow Transplantation, Collaborative Innovation Center of Hematology, Soochow University, Suzhou, People’s Republic of China; 3 Hematology Department, Yancheng Third People’s Hospital, Yancheng, People’s Republic of China

**Keywords:** Multiple myeloma, Minimal residual disease, Next-generation sequencing, IGH gene rearrangement, Next-generation flow cytometry

## Abstract

**Purpose:**

To evaluate the efficacy of next-generation sequencing (NGS) in minimal-residual-disease (MRD) monitoring in Chinese patients with multiple myeloma (MM).

**Methods:**

This study analyzed 60 Chinese MM patients. During MRD monitoring in these patients’ post-therapy, clonal immunoglobulin heavy chain (IGH) rearrangements were detected via NGS using LymphoTrack assays. MRD monitoring was performed using NGS or next-generation flow cytometry (NGF), and the results were compared. Additionally, the sensitivity and reproducibility of the NGS method were assessed.

**Results:**

The MRD detection range of the NGS method was 10^–6^–10^–1^, which suggested good linearity, with a Pearson correlation coefficient of 0.985 and a limit of detection of 10^–6^. Intra- and inter-assay reproducibility analyses showed that NGS exhibited 100% reproducibility with low variability in clonal cells. At diagnosis, unique clones were found in 42 patients (70.0%) with clonal IGH rearrangements, which were used as clonality markers for MRD monitoring post-therapy. Comparison of NGS and NGF for MRD monitoring showed 79.1% concordance. No samples that tested MRD-positive via NGF were found negative via NGS, indicating the higher sensitivity of NGS. MRD could be detected using NGS in 6 of 7 samples before autologous hematopoietic stem-cell transplantation, and 5 of them tested negative post-transplantation. In contrast, the NGF method could detect MRD in only 1 sample pre-transplantation.

**Conclusion:**

Compared with NGF, NGS exhibits higher sensitivity and reproducibility in MRD detection and can be an effective strategy for MRD monitoring in Chinese MM patients.

**Supplementary Information:**

The online version contains supplementary material available at 10.1007/s12672-024-00938-w.

## Introduction

Multiple myeloma (MM) is a clonal plasma-cell tumor, with high cytogenetic heterogeneity [1]. It is the second most common hematological malignancy in many countries [2–4] and has a substantial impact on public health. The specific incidence of MM in China is not clear, but the median age of onset is 57.9–59 years old [5, 6], which is far lower than that observed in western countries [7–9]. In recent years, new chemotherapies and immune drugs have greatly improved the remission rate and survival time in MM [4]. However, as the long-term survival rate of patients has increased, the recurrence rate has also risen, resulting in eventual relapse for the majority of patients, for whom there is currently no cure [10]. Currently, the treatment goals in MM prioritize long-term disease control, disease remission, and improvement of the life quality of the patient. Consequently, there is an urgent need for improved curative outcomes. Additionally, there is an urgent need for sensitive methods for continuous monitoring of minimal residual disease (MRD) and guiding clinical treatment following complete remission [11].

MM represents a group of malignant tumors originating from B lymphocytes. During the normal formation of B cells and their differentiation into plasma cells, recombination of variable (V), diversity (D), and joining (J) gene segments leads to immunoglobulin heavy chain (IGH) rearrangements. The diversity, randomness, and arbitrary insertion and deletion of nucleotides in the junction region during IGH rearrangements result in each B-cell clone having a unique VDJ sequence resulting from its IGH rearrangement [12]. Therefore, the clonal IGH rearrangement identified at the time of diagnosis can serve as a valuable clonal biomarker for MRD monitoring during clinical treatment.

The capillary electrophoresis (CE) method, which has gradually become a reference standard for detecting gene rearrangements, particularly for detecting clonal IGH rearrangements, can be used as an auxiliary diagnostic tool for lymphoid tumors. Yet, its application for MRD monitoring is limited [12]. In contrast, the next-generation sequencing (NGS) technology offers multiple advantages, such as being high-throughput as well as highly time-effective, accurate, and informative. These qualities make NGS highly beneficial in accurately detecting and quantifying clonal rearrangements, especially in the context of MRD monitoring in MM patients post-treatment [13].

The International Myeloma Working Group (IMWG) recommends next-generation flow cytometry (NGF) and NGS as the main methods for evaluating MRD [14]. However, the sensitivity, repeatability, and standardization of clinical correlation analysis of these technologies are still under exploration [15, 16]. In addition, NGS is rarely used to detect MRD in MM patients in China. Thus, this study aimed to assess the applicability of NGS versus NGF to detecting clonal IGH rearrangements in newly diagnosed MM patients and to MRD monitoring post-treatment.

## Methods

### Patients

This retrospective study included 60 newly diagnosed MM patients from the First Affiliated Hospital of Soochow University between September 2019 and October 2020. All the samples from these patients were collected from the biological sample bank used in a previous study [17], during which the patients were treated with bortezomib, lenalidomide, and dexamethasone with or without subsequent autologous stem-cell transplantation (ASCT).

This study was approved by the Ethics Committee of the First Affiliated Hospital of Soochow University and followed the Declaration of Helsinki. Informed consent was obtained from each patient.

### Cytogenetic analysis

At diagnosis, interphase fluorescence in situ hybridization (FISH) analysis was performed alongside sorting CD138 plasma cells. The following probes were used: 1q21 for 1q gain, RB1 and D13S319 for 13q deletion or monosomy 13, TP53 for TP53 deletion or monosomy 17, IGH for 14q32 rearrangements, and probes targeting the individual IGH rearrangements t (11;14)(q13; q32) CCND::IGH, t (4;14)(p16; q32) FGFR3::IGH, and t (14;16)(q32; q23) IGH::MAF. The probes were obtained from GP Medical Technologies, Beijing, China. A minimum of 200 interphase nuclei obtained from bone-marrow (BM) cultures were analyzed using a Leica DMRXA fluorescence microscope (Leica, Wetzlar, Germany).

### DNA extraction

Genomic DNA was isolated from BM aspirates at the time of diagnosis and the follow-up period by using a DNA Extraction Kit (Promega, USA). DNA quantity and quality were evaluated using a Qubit instrument (Thermo Fisher, USA) according to the manufacturer’s instructions.

### PCR and CE

PCR and CE were used to detect IGH clonality [18]. In brief, the IGH-FR1, FR2, and FR3 gene rearrangements were amplified using PCR with the IdentiCloneTM rearrangement detection kit (Invivoscribe, USA) according to the manufacturer’s instructions. An ABI3730 gene analyzer (Thermo Fisher, USA) was used to analyze the amplicons, following previously described scoring criteria [19].

### NGF

The flow-cytometry-based strategy used in this study involved 10 characteristic antibodies, including 8 against cell-membrane proteins (CD138-APC, CD38-APC750, CD19-ECD, CD45-KO, CD56-PC7, CD27-PB, CD81-APC700, and CD117-PC5; from Beckman Coulter, USA), and 2 against cytoplasmic proteins (Kappa-FITC and Lambda-PE; from Dako, Denmark), which have previously been described to be able to differentiate between abnormal and normal plasma cells via flow cytometry [20–22]. Sample preparation and detection were performed as previously described [17]. Briefly, 200 μL–5 mL BM aspirates [(2–20) × 10^6^ cells] were stained with the monoclonal antibodies for 30 min at 20–30 °C after lysing red-blood cells by using ammonium chloride and then washed with phosphate-buffered saline. For intracellular light-chain evaluation, cells were stained with anti-kappa and anti-lambda antibodies after adding membrane breakers, followed by washing and incubation. Flow-cytometry events were acquired and analyzed using a Navios Flow cytometer (Beckman Coulter, USA). One million nucleated cells were obtained each time, and if ≥ 20 abnormal plasma cells were detected, the result was considered positive.

### Clonality testing via NGS

NGS-based clonality testing was performed using commercially available LymphoTrack assays (Invivoscribe, USA) targeting IGH-FR1, IGH-FR2, and IGH-FR3. The assays were performed following the manufacturer’s instructions. PCR amplification was performed using a master mix containing primers that had barcoded sequence adaptors. After the PCR products were purified and quantified, they were sequenced using an Ion S5 sequencer platform (Thermo Fisher, USA). Sequencing data in FASTQ format were analyzed using the LymphoTrack software package (InVivoScribe Technologies, San Diego, CA) [16, 23].

### Criteria for clonality and MRD

The criteria for determining the IGH clonality in newly diagnosed MM patients were as follows: a minimum of five identical sequences obtained through sequencing constituted a clone, and the frequency of the clone needed to be > 5% to be used as a marker for MRD tracking in MM patients, as previously described [13]. All the sequences identified in BM samples from patients in the remission stage were compared with the clonal sequences derived from the tumor cells in the newly diagnosed samples, serving as index clonal sequences. Samples were considered MRD-positive if the same sequences were detected [24].

### Validation of the NGS-based method

To assess the performance characteristics of the NGS-based method, a series of experiments were performed. Commercially available assays containing a positive clonal control (IVS0019) and a polyclonal negative control (IVS0000) were used to prepare successive dilutions of DNA, spanning the range of 10^–6^–10^–1^, to determine the limit of detection of the method. To assess the specificity of this method, patients exhibiting clonality were identified, providing specific clone information. Three samples with different MRD levels were selected. Accordingly, corresponding patients P1, P2, and P3 had MRD levels of 10^–2^, 10^–3^, and 10^–4^, respectively. The NGS results of these three samples were detected before with definite results, and the selection criteria were to cover the test range of different orders of magnitude of MRD. These samples were used for assessing inter- and intra-assay reproducibility.

### Statistical analysis

All the data were statistically analyzed using GraphPad Prism version 9.3.1 (https://www.graphpad.com/scientific-software/prism/). Mean values were compared using the independent sample *t*-test and analysis of variance, and rates were compared using the χ^2^ test. Fisher’s exact test was used to test categorical variables, and the Spearman correlation coefficient was used for statistical comparison. A *p*-value < 0.05 was considered to indicate statistical significance. The graphs were generated using GraphPad Prism version 9.3.1 and R version 4.1.0 (R Core Team).

## Results

### Clinical characteristics of the patients

A total of 60 patients, consisting of 33 males and 27 females, with a median age of 57 (39–70) years, were enrolled in this study. The diagnosis of MM was based on the standard criteria established by the IMWG [14]. Among the patients, there were 28 IgG, 12 IgA, 5 IgD, 13 light-chain, and 2 non-secretory myeloma cases. The cytogenetic abnormalities were detected in 40 cases and included t (4;14), t (11;14), t (14;16), 1q21 gain, and 17p abnormalities. Some cases had 2–3 simultaneous abnormalities. Cytogenetic risk stratification was performed according to the Revised International Staging System (R-ISS) [25]. Accordingly, the R-ISS score was 1 in 9 patients, 2 in 39 patients, and 3 in 8 patients. Additionally, 55 patients were at Durie–Salmon stage III at the time of diagnosis. Among the 60 patients, samples were collected from 36 at initial diagnosis and after remission post-induction therapy, with an additional 7 patients undergoing ASCT transplantation. The clinical characteristics of the participants are presented in Table 1.

**Table 1:** Characteristics of the patients

Characteristics	Patients
Gender, male/female	60 (33/27)
Median age, years (range)	56 (39–70)
M protein type	
IgH	
IgG	28
IgA	12
IgD	5
Non-secretory	2
IgL	13
Kappa chain	1
Lambda chain	12
Cytogenetics	
t (4;14)	18
t (11;14)	10
t (14;16)	1
1q21 gain	33
17p abnormalities	7
Cytogenetic risk	
High risk	26
Standard risk	34
DS	
Stage I, II	5 (8.33%)
Stage III	55 (91.67%)
R-ISS^a^	
Stage I	9 (15%)
Stage II	23 (38.33%)
Stage III	28 (46.67%)
Hemoglobin (g/L, $\bar{\text{x}} \pm {\text{s}}$)	93 ± 27
Albumin (g/L, $\bar{\text{x}} \pm {\text{s}}$)	34.1 ± 7.8
LDH, µmol/L (range)	180.8 (93.3–542.1)
Creatinine, µmol/L (range)	129.07 (39.1–650.5)
Calcium, µmol/L (range)	2.35 (1.94–3.63)

*IgH* immunoglobulin heavy chain, *IgL* immunoglobulin light chain, *DS* Durie–Salmon, *R-ISS* revised international staging system, *LDH* lactate dehydrogenase

### Clonality detection by using NGS

**Fig. 1:**
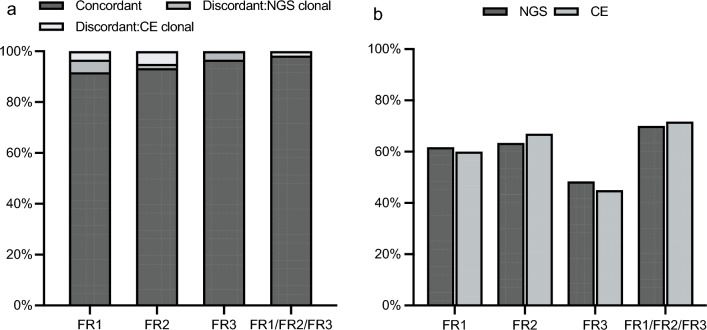
The consistency of NGS and CE in detecting clonal IGH-FR1/FR2/FR3 rearrangements and their detection rates. *NGS* next-generation sequencing, *CE* capillary electrophoresis, *IgH* immunoglobulin heavy chain

All the 60 patients were analyzed using both NGS and CE at the time of diagnosis and tested for FR1, FR2, and FR3 regions simultaneously. The consistency rates for IGH FR1, IGH FR2, and IGH FR3 were 91.7% (55/60), 93.3% (56/60), and 96.7% (58/60), respectively (Fig. 1a). The overall concordance between the two methods was 98.3% (59/60). As shown in Fig. 1b, clonal IGH-FR1, IGH-FR2, and IGH-FR3 rearrangements were detected in 37, 38, and 29 cases via the NGS-based method, respectively, with detection rates of 61.7% (37/60), 63.3% (38/60), and 48.3% (29/60). The overall detection rate of the NGS method was 70.0% (42/60) when considering the combined detection of IGH-FR1/FR2/FR3 rearrangements. The details were described in our previous publication [26].

As shown in Table 2, patients with clonal IGH rearrangements and those with polyclonal rearrangements were compared in terms of the immunoglobulin type, cytogenetic risk stratification, chemotherapy regimen, and ASCT. There was a positive correlation between clonal IGH rearrangement and heavy-chain myeloma cases, including IgG, IgA, IgD, and non-secretory types. No significant difference in distribution was observed for other factors, such as cytogenetics and chemotherapy regimens. Notably, the detection rate of clonal IGH rearrangement significantly differed between the heavy-chain and light-chain myeloma cases (*P* = 0.0012).

**Table 2:** Comparison between the patients carrying clonal IGH rearrangements and those carrying polyclonal rearrangements

Characteristics	Total cases	Clonal^a^	Polyclonal^a^	*P*-value
Ig				0.0012
IgH	47 (78.33%)	38 (63.33%)	9 (15%)	
IgL	13 (21.67%)	4 (6.67%)	9 (15%)	
Cytogenetics^b^				0.3979
SR cytogenetics	34 (56.67%)	22 (36.67%)	12 (20%)	
HR cytogenetics	26 (43.33%)	20 (33.33%)	6 (10%)	
VRd treatment				0.2551
Yes	51 (85%)	34 (56.67%)	17 (28.33%)	
No	9 (15%)	8 (13.33%)	1 (11.11%)	
Transplant				0.7080
Yes	50 (83.33%)	34 (56.67%)	16 (26.67%)	
No	10 (16.67%)	8 (13.33%)	2 (3.33%)	

*IgH* immunoglobulin heavy chain, *IgL* immunoglobulin light chain, *HR* cytogenetics high-risk cytogenetics, *SR* cytogenetics standard-risk cytogenetics, *VRd* bortezomib, lenalidomide and dexamethasone

^a^Clonality of IGH gene rearrangement was determined by the NGS method

^b^*HR cytogenetics* were defined as the presence of t (4;14)(p16; q32), t (14;16)(q32; q23), and /or 17p abnormalities. *SR cytogenetics*: cytogenetic/FISH test done and high risk not explicitly mentioned

### Specificity of NGS

The CDR3 sequence detected using NGS was unique in each patient. As shown in Table 3, among the 42 patients with clonal IGH rearrangements (P1–P42) detected using NGS, each had their own unique IGH rearrangement. Additionally, in 3 patients, bi-allelic rearrangement clones, consisting of one productive and one unproductive IGH rearrangement, were detected. Productive IGH rearrangements were detectable in all the 42 newly diagnosed patients. Analysis of the complementarity-determining region 3 (CDR3) of each IGH rearrangement revealed that the CDR3 sequence of each patient was unique and comprised different V, D, and J gene fragments. The median length of the CDR3 region was 48 base pairs (bp), with a range of 27–78 bp, and the median clonal frequency was 22.7% (5.44–88.26%). As expected, polyclonal IGH rearrangements were detected in peripheral blood samples from healthy individuals.

**Table 3:** Cloning features of IGH rearrangement with MM patients

Patient ID	Clonality	CDR3 nucleotide seq	Length (bp)	Productive	V gene	D gene	J gene	Frequency (%)
P1	clone1	GCGAGAGCCGGGCAAGAAGTTGCTTCTCGTTCCTACTACATGGACGTC	48	Yes	IGHV3-33	IGHD2-21	IGHJ6	22.87
P2	clone1	ACAAAAGGGGGTCGATATGGGCAGTGGCGGCGATTTGACTAC	42	Yes	IGHV3-23	IGHD6-19	IGHJ4	30.94
P3	clone1	ACCACAGATAGTAGCTCTCTCTACTAC	27	Yes	IGHV3-15	IGHD6-6	IGHJ4	24.50
P4	clone1	ATTTTTTCGACCGGCGGCGTCCGCTATAACGGTATGGACGTC	42	Yes	IGHV4-39	IGHD3-3	IGHJ6	25.92
P5	clone1	GCAAAAGACTTCTCTTTTCGATATAGCAGCAGCTGGTTCGGGGCATTTGACTCC	54	Yes	IGHV3-43	IGHD6-13	IGHJ4	35.13
P6	clone1	GCACACAGAGGTACGGTAGGAGTAGACCCTGTAGGGGGGTACTTCTACGCTATGGACGTC	60	Yes	IGHV2-5	IGHD4-17	IGHJ6	5.00
P7	clone1	GCACGCCTAGGATATTGCAGTAGTCTCAGTTGTTATCTTGACTAC	45	Yes	IGHV2-5	IGHD2-2	IGHJ4	26.89
P8	clone1	GCACGGATACGTGGAACTGGAGTTAGGGGCTATTACTACAGCTACTATGGTATGGACGTC	60	Yes	IGHV2-70	IGHD1-1	IGHJ6	33.86
P9	clone1	GCATTGAGTAACTGGCGCTTCTTCTTCGACTAC	33	Yes	IGHV1-3	IGHD1-20	IGHJ4	63.78
P10	clone1	GCCAGATTGGCGCCTACGGGCAACTGGTACTTCGACCTC	39	Yes	IGHV1-69	IGHD4-17	IGHJ2	88.26
P11	clone1	GCGAAAGCCTTCCCGCACGAATTTTTATGGGTGTTCTGGTTGCTAGTCGGAGCTTCAGGTG	61	No	IGHV7-4-1	IGHD1-26	IGHJ1	23.94
	clone2	GTTACAACAGTGGCTGCGGGCGGGCAGTTTGACTCC	36	Yes	IGHV5-51	IGHD6-19	IGHJ4	5.44
P12	clone1	GCGAAAGCGAGCGGATATTGTGATAGTATCAGCTGCCATTTCCTCTTTGACTAC	54	Yes	IGHV3-23	IGHD2-2	IGHJ4	51.67
P13	clone1	GCGAAGGGGACGGGTGCTTATGACCTG	27	Yes	IGHV3-23	IGHD3-10	IGHJ3	44.92
P14	clone1	GCGAGAATCGGGGCGATGGTAGGAACTACTGACCAC	36	Yes	IGHV3-48	IGHD1-7	IGHJ4	27.21
P15	clone1	GCGAGACAATATATGAGTTCGTTGAACTGGTTCAACCCC	39	Yes	IGHV4-39	IGHD3-16	IGHJ5	56.76
P16	clone1	GCGAGACACATTGTTATCATACGAGCCGGCATGACGAGTGTTTACTACTACCTGGACGTC	60	Yes	IGHV4-59	IGHD3-3	IGHJ6	65.22
P17	clone1	GCGAGACAGGGTGGGACGCCTACATCGGACTTCTACTACTACGGTTTGGACGTC	54	Yes	IGHV3-30	IGHD1-26	IGHJ6	40.32
	clone2	GCGAGAGACGGAGTGAGGATCTGGTGACACTTACATTACAGCCGTAAGGGCGTC	54	No	IGHV4-39	IGHD2-21	IGHJ6	9.49
P18	clone1	GCGAGACATGGTTATTACTTTGACAATACTGCTACGTTTGACTAT	45	Yes	IGHV4-39	IGHD3-22	IGHJ4	45.73
P19	clone1	GCGAGACCCCCACCCACGGTCTCTCGAGACTGGTATTTCGATCTC	45	Yes	IGHV3-7	IGHD4-17	IGHJ2	15.53
P20	clone1	GCGAGAGAATATCGGGCCACAGCTGGCGCAGCCTACTCCTTCTACGGTATGGACGTC	57	Yes	IGHV3-33	IGHD6-13	IGHJ6	69.96
P21	clone1	TCCACTGTTTGGATGTCCGACATAGAAGGCACGATTACCCGACGTGACCTC	51	Yes	IGHV3-15	IGHD5-12	IGHJ1	77.58
P22	clone1	GCGAGAGACTTGCTTCCGGGCGAGAGGTTCGGGGAGTGGCCCCCCACCTCCTATCACTACTACTACGGTATGGACGTC	78	Yes	IGHV1-8	IGHD3-10	IGHJ6	60.16
P23	clone1	GCGAGAGAGTTCTGGTACTTTGGGAGTTATTCTCCGCACTACTTCTACGGTATGGACATC	60	Yes	IGHV1-2	IGHD3-10	IGHJ6	43.00
P24	clone1	GCGAGAGATCGGGCCCCGGAAATTCGCGGAGTTCTGATAATAAATGATGACTTT	54	Yes	IGHV1-18	IGHD3-10	IGHJ4	26.37
P25	clone1	GCGAGAGCGTCTCTATTATGGGGATATTGTAGTAGTAGCAGCTGCTCCCTGCCGACCCCTATGGACGTC	69	Yes	IGHV4-34	IGHD2-2	IGHJ6	69.70
P26	clone1	GCGAGAGGCCCGCCAGTACAATATTGTAGTATCACCAGTTGTTATTTGTACCACTTAGACCAC	63	Yes	IGHV3-30	IGHD2-2	IGHJ4	69.33
P27	clone1	GCGAGAGGCGGAGGGCGAAATTACTATTACTACTTCCACATGGACGTC	48	Yes	IGHV7-4-1	N/A	IGHJ6	63.88
P28	clone1	GCGAGAGGCTCGAGAGGATATCTTTTTGACGAGCCAAATTCTAGGCCTCTTATCTACTATTATATAGACGTC	72	yes	IGHV4-59	IGHD2-2	IGHJ6	37.34
P29	clone1	GCGAGAGGTCGCGGATACTGTGATGGCGGTTACTGCACCTCGCGGGCCCCCTACACTCTAGACGTC	66	Yes	IGHV3-11	IGHD2-21	IGHJ6	59.02
P30	clone1	GCGAGAGTGCGGGATTCTGAGTTCTACTTTTTTGACTTC	39	Yes	IGHV3-21	IGHD1-26	IGHJ4	78.01
P31	clone1	GCGAGAGTGTTCGATAGTAGTGGTCTCTATTTCATCGCTTTTGACTCC	48	Yes	IGHV3-30	IGHD3-22	IGHJ4	66.98
P32	clone1	GCGAGCTGTGGTACTCCCAGCTGCTTTCTCGCCGCCCACCAT	42	Yes	IGHV3-21	IGHD2-2	IGHJ4	7.21
P33	clone1	GCGAGGGGGGGGACTCAGTGGAACGGCTATCTTGACTCC	39	Yes	IGHV1-18	IGHD1-1	IGHJ4	64.85
P34	clone1	GCGAGTTCCGTAGGCCACTATAAGTATTGGAGTGGTTATTTGAATTAC	48	Yes	IGHV5-51	IGHD3-3	IGHJ4	60.00
P35	clone1	GCGCGACATTCGCGGATTTATAACTGGTTCGACCCC	36	Yes	IGHV4-59	IGHD3-3	IGHJ5	31.29
P36	clone1	GCGCGCGACGGGGGAGTTCATTATCCTAAATTCTCCCACCACGGAATGGACGTC	54	Yes	IGHV1-18	IGHD3-16	IGHJ6	35.80
P37	clone1	GCGCGGATCTCAAAACTAAGGGGATATAGACACCTGCGTAGAGACTCCCTTTGGTACTACTTCTTCGGTATGGACGTC	78	Yes	IGHV2-70	IGHD5-12	IGHJ6	18.55
	clone2	TTTGCGACTGTGACTTTATGGTGGTGACAACTGACGCCCGTGTTGACTCG	50	No	IGHV1-46	IGHD2-21	IGHJ4	15.00
P38	clone1	GCGGTGCGCAGGATGGATACTAGTGGTTGGTATCGGGGTTTTGACTCC	48	Yes	IGHV5-51	IGHD3-22	IGHJ4	14.89
P39	clone1	GTGAAAGATTGGGGACCCTACGGTGACTCTACCCGTGGAGACGTC	45	Yes	IGHV3-23	IGHD4-17	IGHJ6	7.35
P40	clone1	GTGAGAGACAACATTGTGGCGGTGACAGCTTTTCACCTGGACAATAAGGACGATGCTTTTGAAATA	66	Yes	IGHV1-18	IGHD2-21	IGHJ3	22.35
P41	clone1	GTGAGAGATTACGCCTGGGCCTTCGACACC	30	Yes	IGHV3-48	IGHD3-16	IGHJ5	26.29
P42	clone1	GTGGACCCGGGATGGGGTCTTCAGTCCACTGTGGACGTC	39	Yes	IGHV3-30	IGHD3-16	IGHJ6	49.06

### The limit and linearity of detection of NGS

**Fig. 2:**
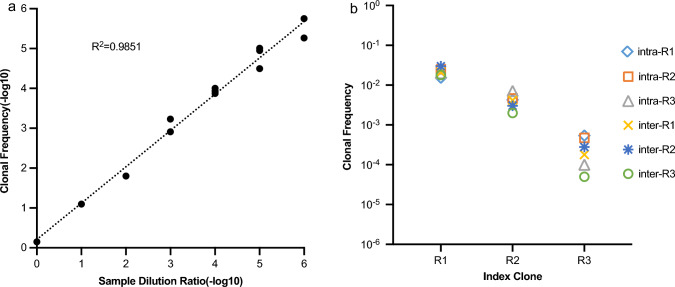
Performance of NGS in detecting clonal IGH rearrangements

To assess the sensitivity of NGS, serially diluted clonal lymphoid DNA was prepared. The IGH rearrangement clones with serial dilutions were detected as the results of MRD. As shown in Fig. 2a, the index clonal sequence was detectable at all the dilution levels and showed a good linear relationship. When the total input was increased to 3 μg DNA in three replicates (1 μg each), the index clonal sequence remained detectable down to 10^–6^ dilution.

### Reproducibility of NGS

Three samples with different MRD levels, previously detected with definitive results, were selected to detect cells of interest at a 0.01% level. Each sample was tested in triplicate to evaluate inter- and intra-assay reproducibility. The results corresponding to the index clone sequence are shown in Fig. 2b. The results within each triplicate were highly consistent, with the index clone being detectable at 10^–2^, 10^–3^, and 10^–4^ dilutions. Clonal cells exhibited 100% reproducibility with low variability (6.13–10.85% coefficient of variation). These findings indicate good reproducibility of NGS in detecting MRD.

### MRD monitoring of MM patients during the follow-up period

**Fig. 3:**
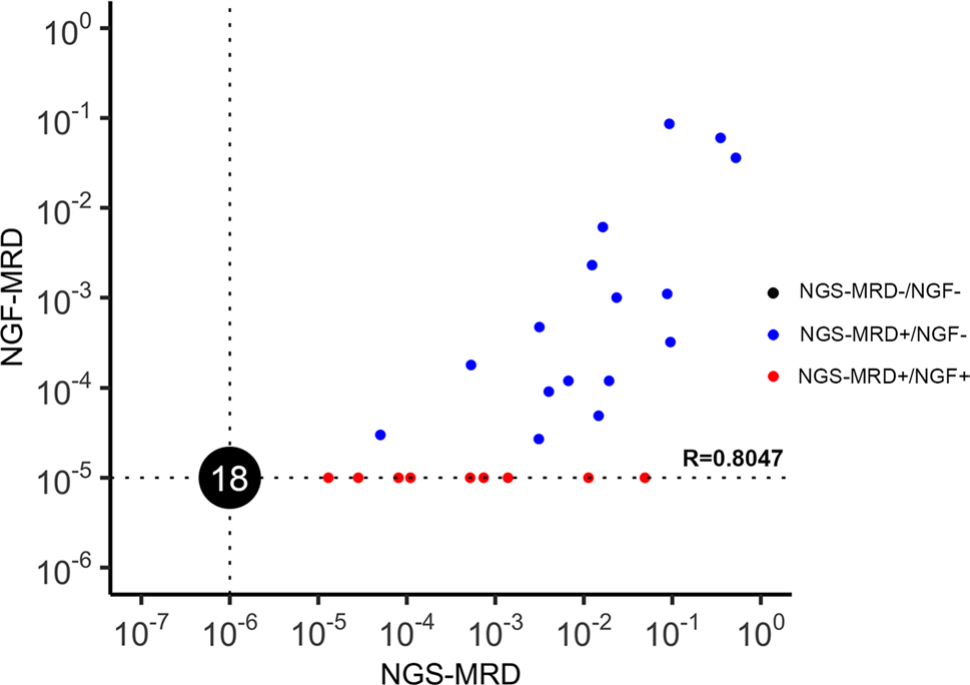
Comparison of NGS (X-axis, assessed at 10^–6^) and NGF (Y-axis, assessed at 10^–5^) in detecting MRD during the follow-up period. The 43 tested samples were classified into four quadrants. The upper-left, upper-right, lower-left, and lower-right quadrants represent the cases tested MRD-positive via NGF but not NGS, via both methods (16 samples), via neither method (18 samples), and via NGS but not NGF (9 samples), respectively

After verifying the performance of NGS in detecting clonal IGH rearrangements, MRD was monitored in 36 patients by using NGS in parallel to NGF. A total of 43 samples from these patients, collected at different time points during MRD monitoring, were tested using both NGS and NGF. Figure 3 shows the comparison of the results derived from the NGS-based analysis (X-axis, assessed at 10^–6^) and those from the NGF-based one (Y-axis, assessed at 10^–5^) in 43 samples from MM patients during the follow-up period. The overall consistency between the two methods was 34/43 (79.1%). The levels of MRD determined using NGS were highly correlated with those determined using NGF (Spearman coefficient *R* = 0.8047). Specifically, all the 16 samples identified as MRD-positive via NGF were also detected as positive via NGS. However, 9 samples that were detected as MRD-positive via NGS were negative according to NGF results. The remaining 18 samples tested MRD-negative via both methods.

Among these patients, 36 were followed up to the remission stage after induction therapy, and 7 of these 36 patients underwent ASCT. MRD detection was performed on samples from these 7 patients before and after ASCT. Details about these 7 patients are shown in Table 4. Among the 7 samples taken before ASCT, only 1 sample tested MRD-positive via NGF. In contrast, 6 of these 7 samples tested positive via NGS. Of these 6 patients, 4 tested negative via NGS after ASTC, and the remaining 2 patients were still positive, indicating a reduction in the number of MRD-positive patients relative to the number before ASTC. Notably, all the post-ASCT samples tested negative via NGF.

**Table 4:** MRD monitoring in MM patients post-ASCT

NO	Sex	Age	Type	DS	ISS	R-ISS	Cytogenetics	Induction regimen	After induction			After ASCT		Outcome
									Response	NGF-MRD	NGS-MRD	NGF-MRD	NGS-MRD	
P1	F	46y	IgA-κ	IIIA	II	II	t(4;14),1q21+	VRd	sCR	0	8.06E−05	0	0	Alive at 24mo
P2	F	46y	λ	IIIA	III	II	t(11;14),1q21+	VRd	sCR	0	7.39E−04	0	0	Alive at 34mo
P3	F	62y	λ	IIIB	III	III	1q21+	VRd	sCR	0	1.39E−03	0	0	Alive at 19mo
P4	F	47y	IgG-λ	IIIA	II	II	Normal	VRd	VGPR	0	1.13E−02	0	2.83E−05	Alive at 18mo
P5	F	58y	IgA-λ	IIIA	I	I	1q21+	VRd	sCR	0	0	0	0	Alive at 20mo
P6	F	64y	IgG-λ+λ	IIIA	II	II	t(4;14),1q21+	VRd	sCR	0	5.20E−04	0	0	Alive at 14mo
P7	M	39y	IgG-λ	IIIA	I	II	t(4;14),1q21+	VRd	VGPR	1.80E−04	5.32E−04	0	1.10E−04	Alive at 12mo

*DS* Durie–Salmon, *ISS* international staging system, *R-ISS* revised international staging system, *ASCT* autologous stem cell transplant, *VRd* bortezomib, lenalidomide and dexamethasone, *sCR* stringent complete response, *VGPR* very good partial response

## Discussion

Therapeutic approaches for treating MM have advanced to include novel drugs, particularly immunotherapies. The combined use of proteasome inhibitors, thalidomide analogs, and CD38-targeting monoclonal antibodies currently represents the mainstay of modern myeloma therapy. New monoclonal antibodies, T-cell activators, and cell therapy are also in the process of entering the clinics. Although a definite cure for MM is still lacking, the introduction of new drugs with different mechanisms and improved treatment approaches has significantly improved the survival of MM patients [27]. MRD has a strong predictive value in various disease states and treatment conditions [28, 29]. It can identify the likelihood of relapse and enable early intervention. Evaluation of MRD rates is also used as an endpoint to accelerate drug testing and approval in many trials [30, 31].

Although many methods, such as NGF and multi-parametric flow cytometry, can be used to detect MRD, there is no standard method. At present, NGF is the most common method used for detecting MRD in clinics. MRD detection based on NGF is fast, efficient, and economical; however, it requires complex visualization and professional data analysis. Furthermore, false-negative MRD detection can occur in some patients due to immunophenotypic changes post-treatment [32].

The use of NGS for detecting MRD has increasingly been implemented in clinical practice. A study has compared NGS and NGF in detecting MRD and concluded a strong correlation between the two approaches [33].

MM patients in China tend to be younger than those in Europe or the United States. Furthermore, there is a high demand for effective management of MM in this population. However, the detection of MRD in Chinese MM patients has primarily relied on flow cytometry, which is gradually becoming insufficient to meet patient needs. Although international studies have previously reported on MRD monitoring via second-generation sequencing, there is limited research on the Chinese population.

To the best of our knowledge, this study is the first to compare NGS with NGF in China. To monitor MRD by using NGS, the clonal IGH rearrangement at the time of diagnosis must be known. In this study, both NGS and CE were used to detect the clonal IGH rearrangements in 60 newly diagnosed Chinese MM patients, and the consistency between the two methods was 98.3%. The overall detection rate of the IGH-FR1/FR2/FR3 combination was 70.0% via NGS. Additionally, unique clonal IGH rearrangements were observed in 42 patients. Therefore, NGS could detect clonal rearrangements in most of the newly diagnosed MM patients. Such detection can serve as a molecular biomarker at the time of diagnosis, enabling MRD monitoring during clinical treatment. To evaluate the feasibility of NGS in follow-up MRD monitoring of MM patients, we analyzed the limit and repeatability of NGS in detecting MRD. According to the IMWG guidelines, MM patients are considered MRD-negative if there are no clonal plasma cells in the bone marrow, with a minimum sensitivity of 1 in 10^5^ nucleated cells via the NGS method [14]. The results of the study presented here confirmed that NGS has good sensitivity in MRD detection, and demonstrated a linear curve ranging from 10^–6^ to 10^–1^, with a correlation coefficient of 0.985. Using this method, it is possible to detect one tumor plasma cell in 1,000,000 nucleated cells, indicating a limit of detection of 10^–6^. Thus, NGS exhibits high sensitivity in MRD detection in MM patients. In addition, this approach showed good repeatability in MRD detection in these patients. In samples with different tumor loads, the MRD levels were estimated at 10^–2^, 10^–3^, and 10^–4^ via NGS, and the intra- and inter-assay variation was relatively low.

Currently, the major approaches recommended for MRD assessment in MM patients at home and abroad are the multi-parameter NGF and NGS technologies. There are relatively many reports on multi-parameter NGF in MRD detection in MM patients [34, 35], whereas the applicability of NGS has seldom been reported in China yet. In this study, 43 samples from 36 patients were evaluated at follow-up by using both NGS and NGF. Our results revealed a consistency rate of 79.1% between the two methods, showing that both methods have high consistency. Interestingly, out of the cases analyzed, 9 showed inconsistent MRD results, with MRD levels being detectable via NGS but undetectable via NGF. It is worth noting that none of the samples identified as MRD-positive via NGF were found to be negative via NGS. Discrepancies between NGF and NGS in detecting MRD can be attributed to differences in sensitivity and detection principles. NGF, which relies on antibodies targeting cell surface proteins, and NGS, which identifies genetic mutations, focus on distinct biological markers [36, 37]. This divergence in methodological focus can lead to scenarios where MRD is detectable by one technique but remains undetected by the other, reflecting the distinct detection capabilities inherent to each method [38].

MM patients undergoing CAR-T therapy targeting MM surface antigens, such as CD138 and CD229, may experience blocking of these antigen-binding sites for several months [39], This necessitates adjustments in the use of NGF for MRD detection. Interestingly, after ASTC, 2 patients tested MRD-negative via NGF. However, the NGS method revealed MRD levels of 2.83 × 10^–5^ and 1.10 × 10^–4^ in these patients. Notably, both patients demonstrated a very good partial response according to the evaluation of treatment effectiveness following treatment with VRd. Previous studies have also reported [40] that after induction treatment or transplantation, MRD that turns negative indicates a better clinical prognosis.

Retrospective studies have shown that making treatment decisions based on MRD results (including stopping, intensifying, or changing the treatment) can improve progression-free survival in comparison with patients whose treatment remains unmodified after MRD assessment [31, 32, 41]. The prognostic value of MRD, as determined by NGS, offers a robust basis for informed treatment adjustments, encompassing de-escalation, intensification, or modification strategies to halt disease progression and improve outcomes [29, 41]. Furthermore, by uncovering the genetic and immunologic drivers of MRD, NGS facilitates the development of targeted therapies, advancing personalized medicine in MM. This transformative approach not only promises improved therapeutic efficacy and patient well-being but also significantly shifts the MM management paradigm [41]. Although NGS presents a higher per-sample costs, its superior sensitivity in detecting MRD at very low levels offers potential long-term cost savings. Early and accurate MRD detection can guide more effective treatment adjustments, potentially reducing the overall treatment costs by avoiding unnecessary therapies and hospitalizations.

Emerging technologies, especially the integration of artificial intelligence (AI) and machine learning (ML) are set to enhance MRD detection in MM by processing complex datasets more efficiently, automating the identification of novel MRD markers, and enabling personalized treatment plans through predictive modeling [42, 43]. Concurrently, the discovery of new biomarkers such as extracellular matrix proteins, angiogenic factors, p53-related protein kinase, circulating tumor cells, and microRNAs is redefining MM diagnosis and treatment [44–48]. The future of MM management is geared towards integrating these technologies and biomarkers into a personalized, predictive, and patient-centered care framework.

## Limitation

The present study has several major limitations, including its retrospective design, the small number of cases and the intrinsic differences between two methods. The sample size was largely dependent on the number of suitable samples available from the biological sample bank, which had been collected from patients treated in a previous study within a specific timeframe. Additionally, the follow-up period was relatively short. In future studies, we intend to address these limitations by expanding the sample size, extending the follow-up duration, and conducting analyses of overall or progression-free survival. It is crucial to conduct prospective studies to confirm the feasibility and utility of NGS in MRD monitoring. Acknowledging the limitations of NGS and NGF technologies is crucial in advancing MRD detection in MM. Issues such as sample quality, with DNA integrity vital for NGS and cell viability for NGF, can impact sensitivity and accuracy, while potential contamination during processing may lead to false positives, affecting clinical decisions [36, 49]. Strict lab protocols help mitigate contamination risks. NGS’s susceptibility to errors in complex genomic regions and NGF’s potential to miss MRD due to antigenic shifts post-treatment highlight inherent technology limitations.

## Conclusion

NGS can be used to detect clonality of IGH rearrangements in the majority of Chinese patients newly diagnosed with MM. Clonality of IGH rearrangements can be used as a molecular biomarker at the time of diagnosis for MRD monitoring after clinical treatment. NGS can be used to detect MRD with high specificity, sensitivity, and repeatability in Chinese MM patients during the follow-up period. However, the correlation between these experimental results and clinical outcomes remains to be confirmed with more samples in clinical practice.

### Supplementary Information

Below is the link to the electronic supplementary material.

Supplementary file 1 (DOCX 15 KB)

Supplementary file 2 (DOCX 21 KB)

## Data Availability

All data included in this study are available upon request by contact with the corresponding author.
