# Supplementary material for: Evaluation of next-generation sequencing versus next-generation flow cytometry for minimal-residual-disease detection in Chinese patients with multiple myeloma
Source: Discov Oncol. 2024 Mar 19;15:78. doi: 10.1007/s12672-024-00938-w (PMC10951185; doi:10.1007/s12672-024-00938-w)
Supplement: Supplementary file 1 — (DOCX 15 KB) [file 12672_2024_938_MOESM1_ESM.docx]

**Supplemental Table 1**  Patients’ characteristics

| Characteristics | Patients |
| --- | --- |
| Gender, Male/Female | 60 (33/27) |
| Median age, years (range) | 56 (39-70) |
| M protein type  IgH |  |
| IgG | 28 |
| IgA | 12 |
| IgD | 5 |
| Non-secretory | 2 |
| IgL | 13 |
| Kappa chain | 1 |
| Lambda chain | 12 |
| Cytogenetics |  |
| t (4;14) | 18 |
| t (11;14) | 10 |
| t (14;16) | 1 |
| 1q21 gain | 33 |
| 17p abnormalities | 7 |
| Cytogenetic risk |  |
| High risk | 26 |
| Standard risk | 34 |
| DS |  |
| Stage Ⅰ, Ⅱ | 5 (8.33%) |
| Stage Ⅲ | 55 (91.67%) |
| R-ISS^a^ |  |
| Stage Ⅰ | 9 (15%) |
| Stage Ⅱ | 23 (38.33%) |
| Stage Ⅲ | 28 (46.67%) |
| Hemoglobin（g/L,‾x ±s） | 93±27 |
| Albumin (g/L,‾x ±s） | 34.1±7.8 |
| LDH, umol/L (range) | 180.8 (93.3-542.1) |
| Creatinine, umol/L (range) | 129.07(39.1-650.5) |
| Calcium, umol/L (range) | 2.35(1.94-3.63) |

*IgH,* immunoglobulin heavy chain; *IgL,* immunoglobulin light chain; *DS,* Durie-Salmon; *R-ISS*, Revised International Staging System; *LDH*, lactate dehydrogenase
