# Supplementary material for: Evaluation of next-generation sequencing versus next-generation flow cytometry for minimal-residual-disease detection in Chinese patients with multiple myeloma
Source: Discov Oncol. 2024 Mar 19;15:78. doi: 10.1007/s12672-024-00938-w (PMC10951185; doi:10.1007/s12672-024-00938-w)
Supplement: Supplementary file 2 — (DOCX 21 KB) [file 12672_2024_938_MOESM2_ESM.docx]

**Supplemental Table 2** Cloning features of IGH rearrangement with MM patients

| Patient ID | Clonality | CDR3 nucleotide seq | Length(bp) | Productive | V gene | D gene | J gene | Frequency(%) |
| --- | --- | --- | --- | --- | --- | --- | --- | --- |
| P1 | clone1 | GCGAGAGCCGGGCAAGAAGTTGCTTCTCGTTCCTACTACATGGACGTC | 48 | Yes | IGHV3-33 | IGHD2-21 | IGHJ6 | 22.87 |
| P2 | clone1 | ACAAAAGGGGGTCGATATGGGCAGTGGCGGCGATTTGACTAC | 42 | Yes | IGHV3-23 | IGHD6-19 | IGHJ4 | 30.94 |
| P3 | clone1 | ACCACAGATAGTAGCTCTCTCTACTAC | 27 | Yes | IGHV3-15 | IGHD6-6 | IGHJ4 | 24.50 |
| P4 | clone1 | ATTTTTTCGACCGGCGGCGTCCGCTATAACGGTATGGACGTC | 42 | Yes | IGHV4-39 | IGHD3-3 | IGHJ6 | 25.92 |
| P5 | clone1 | GCAAAAGACTTCTCTTTTCGATATAGCAGCAGCTGGTTCGGGGCATTTGACTCC | 54 | Yes | IGHV3-43 | IGHD6-13 | IGHJ4 | 35.13 |
| P6 | clone1 | GCACACAGAGGTACGGTAGGAGTAGACCCTGTAGGGGGGTACTTCTACGCTATGGACGTC | 60 | Yes | IGHV2-5 | IGHD4-17 | IGHJ6 | 5.00 |
| P7 | clone1 | GCACGCCTAGGATATTGCAGTAGTCTCAGTTGTTATCTTGACTAC | 45 | Yes | IGHV2-5 | IGHD2-2 | IGHJ4 | 26.89 |
| P8 | clone1 | GCACGGATACGTGGAACTGGAGTTAGGGGCTATTACTACAGCTACTATGGTATGGACGTC | 60 | Yes | IGHV2-70 | IGHD1-1 | IGHJ6 | 33.86 |
| P9 | clone1 | GCATTGAGTAACTGGCGCTTCTTCTTCGACTAC | 33 | Yes | IGHV1-3 | IGHD1-20 | IGHJ4 | 63.78 |
| P10 | clone1 | GCCAGATTGGCGCCTACGGGCAACTGGTACTTCGACCTC | 39 | Yes | IGHV1-69 | IGHD4-17 | IGHJ2 | 88.26 |
| P11 | clone1 | GCGAAAGCCTTCCCGCACGAATTTTTATGGGTGTTCTGGTTGCTAGTCGGAGCTTCAGGTG | 61 | No | IGHV7-4-1 | IGHD1-26 | IGHJ1 | 23.94 |
|  | clone2 | GTTACAACAGTGGCTGCGGGCGGGCAGTTTGACTCC | 36 | Yes | IGHV5-51 | IGHD6-19 | IGHJ4 | 5.44 |
| P12 | clone1 | GCGAAAGCGAGCGGATATTGTGATAGTATCAGCTGCCATTTCCTCTTTGACTAC | 54 | Yes | IGHV3-23 | IGHD2-2 | IGHJ4 | 51.67 |
| P13 | clone1 | GCGAAGGGGACGGGTGCTTATGACCTG | 27 | Yes | IGHV3-23 | IGHD3-10 | IGHJ3 | 44.92 |
| P14 | clone1 | GCGAGAATCGGGGCGATGGTAGGAACTACTGACCAC | 36 | Yes | IGHV3-48 | IGHD1-7 | IGHJ4 | 27.21 |
| P15 | clone1 | GCGAGACAATATATGAGTTCGTTGAACTGGTTCAACCCC | 39 | Yes | IGHV4-39 | IGHD3-16 | IGHJ5 | 56.76 |
| P16 | clone1 | GCGAGACACATTGTTATCATACGAGCCGGCATGACGAGTGTTTACTACTACCTGGACGTC | 60 | Yes | IGHV4-59 | IGHD3-3 | IGHJ6 | 65.22 |
| P17 | clone1 | GCGAGACAGGGTGGGACGCCTACATCGGACTTCTACTACTACGGTTTGGACGTC | 54 | Yes | IGHV3-30 | IGHD1-26 | IGHJ6 | 40.32 |
|  | clone2 | GCGAGAGACGGAGTGAGGATCTGGTGACACTTACATTACAGCCGTAAGGGCGTC | 54 | No | IGHV4-39 | IGHD2-21 | IGHJ6 | 9.49 |
| P18 | clone1 | GCGAGACATGGTTATTACTTTGACAATACTGCTACGTTTGACTAT | 45 | Yes | IGHV4-39 | IGHD3-22 | IGHJ4 | 45.73 |
| P19 | clone1 | GCGAGACCCCCACCCACGGTCTCTCGAGACTGGTATTTCGATCTC | 45 | Yes | IGHV3-7 | IGHD4-17 | IGHJ2 | 15.53 |
| P20 | clone1 | GCGAGAGAATATCGGGCCACAGCTGGCGCAGCCTACTCCTTCTACGGTATGGACGTC | 57 | Yes | IGHV3-33 | IGHD6-13 | IGHJ6 | 69.96 |
| P21 | clone1 | TCCACTGTTTGGATGTCCGACATAGAAGGCACGATTACCCGACGTGACCTC | 51 | Yes | IGHV3-15 | IGHD5-12 | IGHJ1 | 77.58 |
| P22 | clone1 | GCGAGAGACTTGCTTCCGGGCGAGAGGTTCGGGGAGTGGCCCCCCACCTCCTATCACTACTACTACGGTATGGACGTC | 78 | Yes | IGHV1-8 | IGHD3-10 | IGHJ6 | 60.16 |
| P23 | clone1 | GCGAGAGAGTTCTGGTACTTTGGGAGTTATTCTCCGCACTACTTCTACGGTATGGACATC | 60 | Yes | IGHV1-2 | IGHD3-10 | IGHJ6 | 43.00 |
| P24 | clone1 | GCGAGAGATCGGGCCCCGGAAATTCGCGGAGTTCTGATAATAAATGATGACTTT | 54 | Yes | IGHV1-18 | IGHD3-10 | IGHJ4 | 26.37 |
| P25 | clone1 | GCGAGAGCGTCTCTATTATGGGGATATTGTAGTAGTAGCAGCTGCTCCCTGCCGACCCCTATGGACGTC | 69 | Yes | IGHV4-34 | IGHD2-2 | IGHJ6 | 69.70 |
| P26 | clone1 | GCGAGAGGCCCGCCAGTACAATATTGTAGTATCACCAGTTGTTATTTGTACCACTTAGACCAC | 63 | Yes | IGHV3-30 | IGHD2-2 | IGHJ4 | 69.33 |
| P27 | clone1 | GCGAGAGGCGGAGGGCGAAATTACTATTACTACTTCCACATGGACGTC | 48 | Yes | IGHV7-4-1 | N/A | IGHJ6 | 63.88 |
| P28 | clone1 | GCGAGAGGCTCGAGAGGATATCTTTTTGACGAGCCAAATTCTAGGCCTCTTATCTACTATTATATAGACGTC | 72 | yes | IGHV4-59 | IGHD2-2 | IGHJ6 | 37.34 |
| P29 | clone1 | GCGAGAGGTCGCGGATACTGTGATGGCGGTTACTGCACCTCGCGGGCCCCCTACACTCTAGACGTC | 66 | Yes | IGHV3-11 | IGHD2-21 | IGHJ6 | 59.02 |
| P30 | clone1 | GCGAGAGTGCGGGATTCTGAGTTCTACTTTTTTGACTTC | 39 | Yes | IGHV3-21 | IGHD1-26 | IGHJ4 | 78.01 |
| P31 | clone1 | GCGAGAGTGTTCGATAGTAGTGGTCTCTATTTCATCGCTTTTGACTCC | 48 | Yes | IGHV3-30 | IGHD3-22 | IGHJ4 | 66.98 |
| P32 | clone1 | GCGAGCTGTGGTACTCCCAGCTGCTTTCTCGCCGCCCACCAT | 42 | Yes | IGHV3-21 | IGHD2-2 | IGHJ4 | 7.21 |
| P33 | clone1 | GCGAGGGGGGGGACTCAGTGGAACGGCTATCTTGACTCC | 39 | Yes | IGHV1-18 | IGHD1-1 | IGHJ4 | 64.85 |
| P34 | clone1 | GCGAGTTCCGTAGGCCACTATAAGTATTGGAGTGGTTATTTGAATTAC | 48 | Yes | IGHV5-51 | IGHD3-3 | IGHJ4 | 60.00 |
| P35 | clone1 | GCGCGACATTCGCGGATTTATAACTGGTTCGACCCC | 36 | Yes | IGHV4-59 | IGHD3-3 | IGHJ5 | 31.29 |
| P36 | clone1 | GCGCGCGACGGGGGAGTTCATTATCCTAAATTCTCCCACCACGGAATGGACGTC | 54 | Yes | IGHV1-18 | IGHD3-16 | IGHJ6 | 35.80 |
| P37 | clone1 | GCGCGGATCTCAAAACTAAGGGGATATAGACACCTGCGTAGAGACTCCCTTTGGTACTACTTCTTCGGTATGGACGTC | 78 | Yes | IGHV2-70 | IGHD5-12 | IGHJ6 | 18.55 |
|  | clone2 | TTTGCGACTGTGACTTTATGGTGGTGACAACTGACGCCCGTGTTGACTCG | 50 | No | IGHV1-46 | IGHD2-21 | IGHJ4 | 15.00 |
| P38 | clone1 | GCGGTGCGCAGGATGGATACTAGTGGTTGGTATCGGGGTTTTGACTCC | 48 | Yes | IGHV5-51 | IGHD3-22 | IGHJ4 | 14.89 |
| P39 | clone1 | GTGAAAGATTGGGGACCCTACGGTGACTCTACCCGTGGAGACGTC | 45 | Yes | IGHV3-23 | IGHD4-17 | IGHJ6 | 7.35 |
| P40 | clone1 | GTGAGAGACAACATTGTGGCGGTGACAGCTTTTCACCTGGACAATAAGGACGATGCTTTTGAAATA | 66 | Yes | IGHV1-18 | IGHD2-21 | IGHJ3 | 22.35 |
| P41 | clone1 | GTGAGAGATTACGCCTGGGCCTTCGACACC | 30 | Yes | IGHV3-48 | IGHD3-16 | IGHJ5 | 26.29 |
| P42 | clone1 | GTGGACCCGGGATGGGGTCTTCAGTCCACTGTGGACGTC | 39 | Yes | IGHV3-30 | IGHD3-16 | IGHJ6 | 49.06 |
